# Exosomes: a promising avenue for cancer diagnosis beyond treatment

**DOI:** 10.3389/fcell.2024.1344705

**Published:** 2024-02-13

**Authors:** Zhu Wang, Qianqian Wang, Feng Qin, Jie Chen

**Affiliations:** ^1^ Breast Center, West China Hospital, Sichuan University, Chengdu, China; ^2^ Department of General Surgery, West China Hospital, Sichuan University, Chengdu, China; ^3^ Institute for Breast Health Medicine, West China Hospital, Sichuan University, Chengdu, China; ^4^ Department of Oncology, The First Affiliated Hospital of Zhengzhou University, Zhengzhou, China; ^5^ School of Basic Medicine, Dali University, Dali, Yunnan, China

**Keywords:** exosome, cancer, diagnosis, therapy, translational research

## Abstract

Exosomes, extracellular vesicles secreted by cells, have garnered significant attention in recent years for their remarkable therapeutic potential. These nanoscale carriers can be harnessed for the targeted delivery of therapeutic agents, such as pharmaceuticals, proteins, and nucleic acids, across biological barriers. This versatile attribute of exosomes is a promising modality for precision medicine applications, notably in the realm of cancer therapy. However, despite their substantial therapeutic potential, exosomes still confront challenges tied to standardization and scalability that impede their practice in clinical applications. Moreover, heterogeneity in isolation methodologies and limited cargo loading mechanisms pose obstacles to ensuring consistent outcomes, thereby constraining their therapeutic utility. In contrast, exosomes exhibit a distinct advantage in cancer diagnosis, as they harbor specific signatures reflective of the tumor’s genetic and proteomic profile. This characteristic endows them with the potential to serve as valuable liquid biopsies for non-invasive and real-time monitoring, making possible early cancer detection for the development of personalized treatment strategies. In this review, we provide an extensive evaluation of the advancements in exosome research, critically examining their advantages and limitations in the context of cancer therapy and early diagnosis. Furthermore, we present a curated overview of the most recent technological innovations utilizing exosomes, with a focus on enhancing the efficacy of early cancer detection.

## 1 Introduction

Cancer, an intricate and pervasive disease, presents a formidable global threat to human life. With a reach that spans across age, gender, and diverse backgrounds, cancer strikes fear into millions of the population (Thill et al., 2023; Wolf et al., 2023). Moreover, cancer places a substantial economic burden on healthcare systems and societies worldwide due to escalating costs associated with treatment and supportive care (Amini et al., 2022; Munshi et al., 2022). What makes cancer particularly challenging is its capacity to infiltrate any organ or tissue, from the lungs to the skin, and from the brain to the bones (Hayashi et al., 2023; Li and Zheng, 2023). Often, its stealthy progression remains concealed until it develops an advanced stage (i.e., usually hard to treat), rendering early detection and treatment a critical challenge (Aleksandrowicz et al., 2023; Wang W T et al., 2023).

Curing cancer is an exceptionally tough endeavor, largely due to the obstacle known as metastasis (Hu Z et al., 2023; Zhou et al., 2023). Metastasis entails cancer cells breaking away from the primary tumor, traversing the bloodstream or lymphatic system, and establishing secondary tumors in distant body parts. This ability to spread and infiltrate new organs or tissues renders cancer highly elusive and resistant to conventional treatment approaches (Hayashi et al., 2023; Li and Zheng, 2023). By the time metastasis is detected, cancer is often at an advanced stage, making complete eradication considerably more challenging. Each secondary tumor presents unique challenges, demanding specific approaches, and the intricate complexity of metastatic disease necessitates a multifaceted treatment strategy (Feng et al., 2023; Gao et al., 2023). Furthermore, cancer cells can evolve, developing resistance to previously effective therapies (Gera et al., 2023; Mahmoud et al., 2023). Therefore, early detection of cancer before its metastasis is critical for cancer treatment and enhances the survival of cancer patients. However, tackling metastasis remains one of the most formidable hurdles on the path to finding a cure for cancer (Wang et al., 2023a; Holladay et al., 2023).

Traditional cancer diagnostic techniques, while historically invaluable, grapple with significant limitations (Xiao, 2015). Many of these methods require invasive procedures like surgeries to obtain tissue samples for analysis, which can be painful and carry risks, making them unsuitable for the patients (Zhang et al., 2013). Additionally, these conventional diagnostic approaches can incur prohibitively high costs, burdening both healthcare systems and patients, particularly when multiple tests are necessary to confirm a diagnosis or monitor treatment progress. Furthermore, their sensitivity, especially in detecting early-stage cancers, can be limited, resulting in smaller tumors or cancerous cells missing until they reach more advanced and less treatable stages (Bassani et al., 2023; Kawada et al., 2023; Tan and Hosein, 2023; Zhang Y et al., 2023). To overcome these limitations, there has been a growing endeavor on developing non-invasive, cost-effective, and highly sensitive diagnostic methods, such as liquid biopsies and advanced imaging technologies (Young et al., 2023). These innovations hold the potential to revolutionize cancer diagnosis by offering earlier detection, reduced invasiveness, and more precise monitoring, ultimately enhancing our ability to combat this devastating disease.

Exosomes have emerged as a burgeoning field of research with vast potential in the realm of cancer detection (Hyun et al., 2018; Xia et al., 2020). These nanoscale particles, once dismissed as cellular debris, are now recognized for their pivotal role in intercellular communication and their capacity to transport molecular cargo, including nucleic acids and proteins (Zhang et al., 2016; Maia et al., 2018). In the context of cancer development, exosomes assume a critical role by ferrying tumor-specific molecules that can serve as biomarkers for early detection (Regev-Rudzki et al., 2013). Their presence in bodily fluids like blood and urine renders them a highly attractive source for non-invasive and easily accessible diagnostic tests (Agnoletto et al., 2023; Wang, et al., 2023b). Researchers are increasingly harnessing the exosomes to detect cancer at its earliest stages, especially before the metastasis when treatment interventions are most effective (Lin et al., 2021; Yu et al., 2021). Furthermore, the diverse array of information conveyed within exosomes offers a plethora of potential markers, enabling not only the detection of cancer but also the identification of specific cancer types and their distinctive molecular characteristics (Sharma and Salomon, 2020). This approach holds significant promise for developing specific treatment strategies for individual patients, thereby improving treatment outcomes and reducing the burden of unnecessary treatments (Kumata et al., 2018; Luo et al., 2018; Smith and Lam, 2018).

In conclusion, the investigation of exosomes as a method for cancer detection marks a promising and innovative Frontier in the field of oncology research (Li G et al., 2020; Zhang W et al., 2021). It holds the tremendous potential to reform the field of early diagnosis, monitoring, and treatment decisions on cancers. As innovative methods continue to emerge in this burgeoning field, exosomes have the potential to become a pivotal element in combatting cancer. They offer patients more efficient and minimally invasive diagnostic alternatives, potentially enhancing treatment efficacy, prolonging survival, and significantly improving the quality of life for those affected by cancer.

## 2 Generation of exosome and its function

**FIGURE 1:**
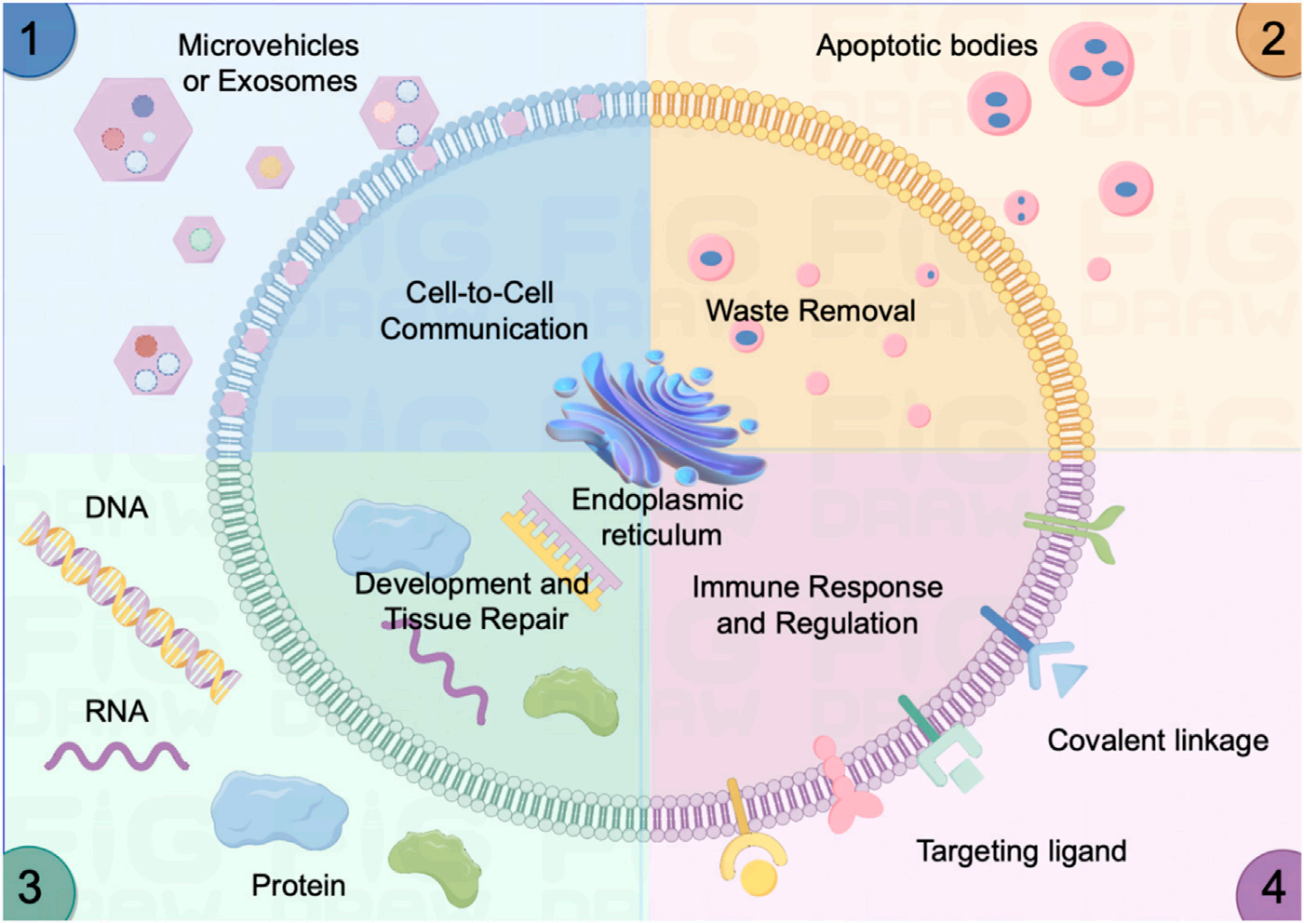
Sketch of the activity and functions of the micro vehicles/exosome *in vivo*. (1) Cell-Cell Communication: Mediate intercellular communication by transporting essential molecules, fostering coordination in physiological processes. (2) Efficient Waste Removal: Serve as carriers for cellular waste removal, contributing to cellular cleanliness (e.g., apoptotic bodies) and overall tissue health. (3) Developmental Regulation and Tissue Repair: Regulate developmental processes and tissue repair by delivering signaling molecules (e.g., DNA, micro RNAs, and proteins) that influence cellular activities. (4) Immune Response Modulation: Actively modulate immune responses by conveying signals via targeting ligand and covalent linkage between immune cells and somatic cells, contributing to inflammation regulation and immune system balance.

Exosomes play a crucial role in cellular quality control, ensuring the maintenance of cellular health. This assures their potential therapeutic applications, especially in conditions marked by impaired waste removal processes (Kakiuchi et al., 2023; Zhao J et al., 2023). The primary functions of exosomes are sketched in Figure 1.

### 2.1 Cell-to-cell communication

Exosomes play a fundamental role in the intricate network of cell-to-cell communication within biological systems. These nanoscale vesicles serve as essential messengers between cells, functioning as conduits for the intercellular exchange of diverse molecular cargo, including proteins, nucleic acids (both DNA and RNA), and lipids (Luo et al., 2023; Shang et al., 2023). When a cell secretes exosomes, they can be internalized by neighboring or distant recipient cells, thereby facilitating the transfer of vital information (Altintas and Saylan, 2023). This molecular exchange enables cells to coordinate and regulate a wide array of cellular activities and responses. For instance, immune cells utilize exosomes to convey signals that orchestrate immune responses, while cancer cells exploit exosome communication to promote tumor growth and metastasis (De La Pena et al., 2009; Kang et al., 2023).

### 2.2 Waste removal

In the network of cellular metabolite maintenance, exosomes serve as indispensable tools for waste removal and cellular quality control mechanisms (Lu et al., 2023). Cells harness the remarkable capacity of exosomes to encapsulate and transport a variety of unwanted or degraded cellular components, such as misfolded proteins, broken organelles, and waste of metabolites, to facilitate their efficient elimination from the cell (Gudbergsson and Johnsen, 2019; Schubert, 2020). This waste disposal process not only ensures the preservation of cellular integrity and functionality but also contributes to the overall health and homeostasis of multicellular organisms. Moreover, the role of exosomes in waste removal extends beyond the individual cell, as they can be released into extracellular environments, such as bodily fluids, where they can aid in systemic waste clearance and potentially play a role in intercellular signaling (Nik Mohamed Kamal and Shahidan, 2021; Lai et al., 2023).

### 2.3 Development and tissue repair

Exosomes also serve as pivotal mediators in critical biological processes, including tissue regeneration and development (Li et al., 2015; Pan et al., 2023). They orchestrate the process of growth and repair in tissues by facilitating the transfer of growth factors and an array of signaling molecules to target cells (Wang and Yang, 2023; Zhao X et al., 2023). In the context of tissue regeneration, exosomes act as specialized messengers, guiding and stimulating cellular responses necessary for the healing and rebuilding of damaged or injured tissues. Notably, in the nervous system, exosomes play an indispensable role in neuronal communication (i.e., synapse) and maintenance (Liu et al., 2022). They serve as nanocarriers, shuttling neurotransmitters, with a myriad of signaling molecules between neurons and other supporting cells within the brain. This intercellular exchange not only underpins fundamental processes in neural development but also contributes to the fine-tuning of neural circuits, highlighting the profound impact of exosomes on neurological function and giving potential therapeutic applications in neurodegenerative diseases and neurological disorders (Hu T et al., 2023; Liu et al., 2023; Zhong et al., 2023).

### 2.4 Immune response regulation

Exosomes assume a crucial role in orchestrating immune responses, wielding their influence in the intricate domain of immune system function (Schwarzenbach and Gahan, 2021). Immune cells harness the power of exosomes to disseminate signaling molecules that serve as directives for the regulation of immune responses, effectively coordinating the complex interplay of immune mechanisms (Greening et al., 2015; Chen et al., 2017; Li et al., 2019). Moreover, exosomes act as couriers of information, transferring crucial insights about pathogens or abnormalities to other immune cells, thereby enabling swift and coordinated countermeasures against infections or aberrant cell behavior (Kaminski et al., 2019; Lema and Burlingham, 2019). In the realm of disease, exosomes take on added significance as potential diagnostic tools. For instance, cancer cells exploit exosomes as conveyors of disease-specific cargo, including tumor-specific proteins and genetic material which further modulate the immune response (Anel et al., 2019; Shen et al., 2019).

**FIGURE 2:**
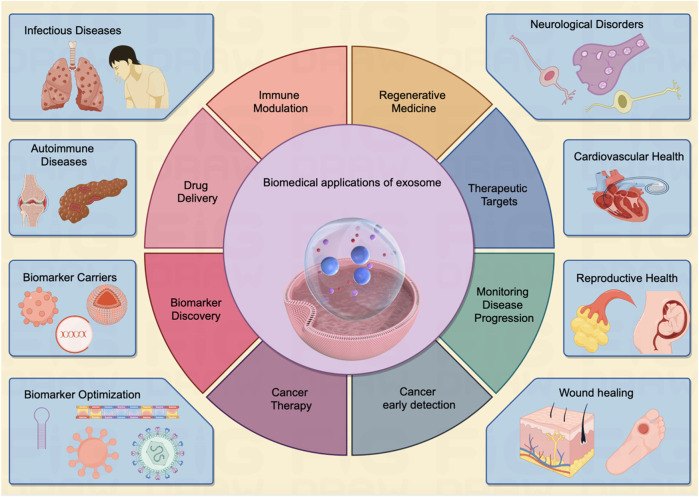
The exosome exerts multi-faceted biomedical potentials for human disease. Exosomes demonstrate a vast array of biomedical applications pertinent to human diseases affecting various organs throughout the body. These entities serve as pivotal biomarkers in diagnostic processes and hold considerable potential across multiple domains, including targeted drug delivery, immunomodulation, and tissue regeneration. As multifunctional agents, they are increasingly acknowledged as instrumental in driving forward the Frontier of innovative biomedical research, heralding a notable shift in the paradigms of disease detection and therapeutic strategies, as illustrated in the center panel. The inherent attributes of exosomes establish them as an emergent and significant field within biomedical research, poised to radically reshape methodologies in disease diagnosis, therapeutic interventions, and the broader spectrum of healthcare practices.

Of note, exosomes have garnered significant attention in the field of cancer research, as they are believed to play a critical role in cancer progression, metastasis, and drug resistance (Lema and Burlingham, 2019; Shen et al., 2019). Researchers are exploring the potential of exosomes as diagnostic markers and therapeutic targets in various diseases, including cancer, neurodegenerative disorders, and autoimmune conditions (Figure 2). Their ability to carry specific molecular cargo makes them a promising avenue for understanding disease mechanisms and developing novel medical treatments (Table 1).

**TABLE 1:** Contents in the exosomes collected for immune response in recent studies.

Sources	Contents	Effect	References
Alveolar epithelial type II cells	Viral particles	Help SARS-CoV for immune system evasion	Qian et al. (2013); Mason (2020)
CD4^+^ T cells	Proteins of HIV	Inhibit and induce the apoptosis of CD4^+^ T cells	Ali et al. (2010), Lenassi et al. (2010), de Carvalho et al. (2014)
Dendritic cells	MHC-I and MHC-II	Associated with T-cell-dependent anti-tumor response	Hsu et al. (2003)
Glioma	DNA	Cross the intact blood-brain barrier and present in peripheral blood	García-Romero et al. (2017)
HIV-infected cells	Viral nucleic acids of HIV	Deliver viral nucleic acids to the health cells	He et al. (2010), Narayanan et al. (2013)
Human breast milk	Oligosaccharides	Decrease the inflammation in the gut	Okoye et al. (2014)
Intestinal epithelial cells	MHC-II and Fas ligand	Associated with the apoptosis of dendritic cells	Takenaka and Quintana (2017)
Intestinal epithelial cells	MHC-II and Fas ligand	Associated with Microglial activation, decline of synaptic stability	Hasegawa and Matsumoto (2018)
Intestinal epithelial cells	Integrin αvβ6	Upregulate the TGF-β in dendritic cells	Hasegawa and Matsumoto (2018)
Lung adenocarcinoma	dsDNA	Double-stranded DNA in exosomes of malignant pleural effusions, biomarkers for lung adenocarcinoma	Loscocco et al. (2019)
Lymphocytes	miR-142-3p, miR-142-5p, and miR-155	Associated with the apoptosis of the beta cells in pancreas	Palmisano et al. (2012)
Macrophages	miRNA-88 and miRNA-99	activate the tumor necrosis factor-alpha in the immune response to HIV infection	Bernard et al. (2014)
Megakaryocytes and platelets	C-X-C chemokine receptor type 4 (CXCR4)	Increase the susceptibility of CXCR4-null cells respond to HIV infection	Rozmyslowicz et al. (2003)
Melanoma	dsDNA	Double-stranded DNA (dsDNA) as a potential biomarker for melanoma detection	Thakur et al. (2014)
NSC-34 motor neurons	miR-124	Induce senescence in microglia cells and reduce phagocytic abilities	Zhao et al. (2019)
Osteosarcoma	Repetitive element DNAs	Potential biomarkers for osteosarcoma	Cambier et al. (2021)
Ovarian cancer	mtDNA	mtDNA in exosomes, biomarkers for ovarian cancer	Keseru et al. (2019)
Regulatory T cells	Let-7d	Suppress the proliferation of Th1 cell	Kurakevich et al. (2013)
Serum	miR-424-5p	Inhibit granulosa cell proliferation and induce cell senescence for the patients with polycystic ovary syndrome	Bozdag et al. (2016)

## 3 The biomedical application of exosome

Exosomes have gained immense significance in the field of biomedicine due to their diverse and versatile applications (Greening et al., 2015). These exosomes carry a variant of molecular cargo, making them valuable tools for numerous biomedical applications (Whiteside, 2017). The key biomedical applications of exosomes are indicated in Figure 2.

### 3.1 Regenerative medicine

Exosomes derived from stem cells contain growth factors and signaling molecules that can stimulate cell proliferation and tissue healing. Exosome-based therapies are being explored for conditions like heart disease and tissue injuries (Zhang and Cheng, 2023). It can be used in more multiple-system disease treatments such as follows:

Neurological disorders: Exosomes are involved in intercellular communication in the nervous system. They may play a role in the pathogenesis of neurodegenerative diseases such as Alzheimer’s and Parkinson’s. Studying exosomes in these contexts can provide insights into disease mechanisms and potential therapeutic interventions (Xiao et al., 2021). Recent studies show that exosomes from neural progenitor cells promote neuronal differentiation and neurogenesis via miR-21a (Ma et al., 2019). Similarly, exosomes from human induced pluripotent stem cell (hiPSC)-derived neurons enhance proliferation in human primary neural cultures *in vitro*. Injection of exosomes from rodent primary neural cultures into P4 mouse brains also increases neurogenesis in the dentate gyrus of the hippocampus (Sharma et al., 2019).

Cardiovascular health: Exosomes are being explored as diagnostic and therapeutic tools in cardiovascular diseases. They can carry molecules associated with heart conditions and may be used to monitor and treat heart diseases (Li N et al., 2023). Wang et al. report that exosomes from induced pluripotent stem cells (iPSCs) transfer cardioprotective miRNAs, including miR-21 and miR-210, to cardiomyocytes. Elevated miRNA levels in iPSC-exosomes contribute to protection against oxidative stress in H9C2 cells by inhibiting caspase 3/7 activation. These anti-apoptotic effects were validated in a mouse model of acute myocardial ischemia/reperfusion (Zhang et al., 2015).

Reproductive health: Exosomes play roles in reproductive biology, including sperm function and embryo development. They may have applications in fertility treatments and the study of reproductive disorders (Ranjbaran et al., 2019; Pu et al., 2022). Zhao et al. explored the impact of exosomal miR-323-3p from adipose mesenchymal stem cells (AMSCs) on cumulus cells (CCs) of polycystic ovary syndrome (PCOS) patients, finding that it inhibited apoptosis by targeting programmed cell death protein 4 (PDCD4) and alleviated PCOS (Zhao et al., 2019). Another study revealed that exosomes from the serum of PCOS patients significantly stimulated the migration and invasion of endometrial cancer cell lines. The study identified miR-27a-5p as highly induced in serum exosomes from PCOS patients and demonstrated its direct targeting of the tumor suppressor gene SMAD4 in the TGF-β signaling pathway (Che et al., 2020).

Wound healing: Exosomes have shown great promise in wound healing due to their ability to carry and deliver bioactive molecules, such as growth factors and microRNAs, to the damaged tissue. These exosomes can promote tissue regeneration, reduce inflammation, and accelerate the overall wound-healing process (Kudinov et al., 2021; Geng et al., 2022). A study engineered MSCs to produce exosomes enriched with long non-coding RNA H19, regulating the PI3K/AKT pathway and suppressing inflammation and apoptosis in a diabetic foot ulcer mouse model (Li B et al., 2020). Additionally, nanoparticles loaded into exosomes, specifically BMSCs-exosomes with magnetic Fe3O4 nanoparticles and increased exosome miR-1260a, were found to boost angiogenesis (Wu et al., 2021).

### 3.2 Immune modulation

Exosomes derived from immune cells play a pivotal role in orchestrating immune responses within the body (Gangadaran et al., 2022b). These tiny vesicles serve as potent messengers of intercellular communication, carrying a cargo of bioactive molecules such as cytokines, chemokines, and antigens (Trams et al., 1981). When immune cells encounter pathogens or foreign invaders, they release exosomes that can activate or suppress immune reactions, depending on the context (Table 1). For instance, dendritic cell-derived exosomes can present antigens to T cells, initiating adaptive immune responses against infections or cancer (Shenoda and Ajit, 2016). Conversely, regulatory T cell-derived exosomes can exert immunosuppressive effects, modulating excessive inflammation and autoimmunity (Arenaccio et al., 2014). Numerous studies highlight the role of CD8^+^ T cell-derived exosomes in facilitating communication between immune cells and tumor cells, regulating tumor development. Fully activated CD8^+^ CTLs release exosomes that boost the activation of low-affinity CD8^+^ T cells, contributing to the tumor-killing process (Li et al., 2017; Wu S W et al., 2019). Understanding the intricate role of immune cell-derived exosomes is crucial for advancing our knowledge of immune regulation and developing novel therapeutic strategies for immune-related disorders.

### 3.3 Drug delivery

Exosomes also have emerged as promising natural nanocarriers for drug delivery in recent years (Gangadaran et al., 2022a). First, their natural origin minimizes immunogenicity and toxicity concerns, making them well-tolerated *in vivo*. Second, exosomes are equipped with cell-targeting proteins and bioactive molecules on their surface, facilitating specific delivery to target cells or tissues (Gangadaran et al., 2022c). Furthermore, their stability in circulation and ability to traverse biological barriers, such as the blood-brain barrier, offer a versatile platform for encapsulating various therapeutic cargoes, including small molecules, nucleic acids, and proteins (Krishnan et al., 2022). Harnessing exosomes as nanocarriers holds great potential to enhance the precision and efficacy of drug delivery in diverse therapeutic applications.

### 3.4 Biomarker discovery for cancer treatment

Exosome cargo analysis can facilitate the discovery of disease-specific biomarkers. These biomarkers can be used for early disease detection, monitoring disease progression, and assessing treatment responses. Of note, the exosome is promising for the cancer diagnostics (Si et al., 2023). Exosomes derived from cancer cells contain specific biomarkers, such as mutant DNA, proteins, and microRNAs, that can serve as indicators of cancer presence and progression (Ji et al., 2013; Miaomiao et al., 2023). Liquid biopsies based on exosome cargo analysis offer non-invasive methods for early cancer detection and monitoring treatment responses (Feng et al., 2019). Moreover, exosome-based diagnostics and therapeutics represent a promising avenue in cancer therapy (Kalluri and LeBleu, 2020). Researchers are exploring the use of exosomes for targeted drug delivery. By engineering exosomes to carry therapeutic molecules, drugs can be directed specifically to cancer cells, reducing off-target effects and improving treatment efficacy (Barile and Vassalli, 2017; Chen H et al., 2021; Jin et al., 2021). Briefly, enhancing exosome targeting for drug delivery platforms involves selecting specific exosome donors or employing bioengineering techniques. This can be achieved by modifying the exosome surface with homing molecules through ligands, magnetic materials, charge affinity, and pH-responsive motifs (Butreddy et al., 2021). Ultimately, by loading the drug into these modified exosomes, targeted carriers can be created for specific cells or organs, leading to improved clinical treatment outcomes (He et al., 2022). The accumulation of drugs in target sites enhances the efficacy of exosomes while simultaneously reducing off-target effects.

Overall, the biomedical applications of exosomes are vast and continually expanding. Their unique properties as carriers of molecular information, combined with their potential for non-invasive sampling and targeted delivery, make exosomes a promising area of research and development in the quest to better understand, diagnose, and treat a wide range of diseases and medical conditions.

## 4 The application of exosomes for cancer

Exosomes play a crucial role in cancer progression and metastasis by facilitating the transfer of bioactive molecules between cancer cells and diverse cells in both local and distant microenvironments. This intercellular communication leads to alterations in various cellular and biological functions within the recipient cells (Jiang et al., 2021). Additionally, exosomes serve as indicators of the heterogeneity present in cancer tumors (Al-Nedawi et al., 2008). Distinct subpopulations of cancer cells within a single tumor release exosomes with unique molecular profiles (Maia et al., 2018). Recognizing and understanding this heterogeneity is essential as it can guide treatment decisions and potentially pave the way for personalized therapy strategies.

### 4.1 Biomarker carriers for early detection of cancers

Exosomes have emerged as a promising Frontier in cancer research and diagnostics, driven by their distinctive cargo (Tai et al., 2018). These microscopic vesicles encapsulate a wealth of information derived from cancer cells, serving as invaluable reservoirs of diverse cancer biomarkers, including genetic mutations, oncogenic proteins, and a spectrum of cancer-specific molecules (Jiang et al., 2021; Liu et al., 2021). The encapsulation of such biomarkers within exosomes is of paramount significance, as they can serve as pivotal indicators of cancer initiation, progression, and response to therapeutic interventions (Table 2).

**TABLE 2:** Partial biomarkers collected for cancer detection in recent studies.

Biomarkers	Cancer type	Signatures	References
Annexins 1	Glioblastoma	Elevated ANXA1 transcript levels were similarly detected in cases of glioblastoma (GBM)	Mallawaaratchy et al. (2017)
Annexins 2	Breast cancer	Overexpression of Annexin A2 has also been confirmed in various malignancies	Maji et al. (2017)
Annexins 6	Breast cancer	Its role varies depending on the type of cancer, as it can function either as a tumor suppressor or as a promoter of motility	Sakwe et al. (2011)
CD151	Lung cancer	Increased CD151 expression in cancer is associated with an unfavorable prognosis	Niu et al. (2019)
CD63	Melanoma	Reduced expression of CD63 upon cancer progression	Pols and Klumperman (2009)
CD81	Breast cancer	CD81 exhibits increased expression within the stromal region linked to human invasive ductal carcinoma	Luga et al. (2012)
CD9	Prostatic carcinoma	Reduced expression of CD9 in metastatic lesions	Wang et al. (2007)
Flotillin2	Prostatic carcinoma	The upregulated expression of flotillin 2 is associated with cancer progression and prognosis	Wang et al. (2017)
GTPase Rab 11	Colorectal cancer	Overexpression of Rab11 is associated with a prognosis	Chung et al. (2014)
GTPase Rab 27	Lung cancer	Downregulated expression of Rab27 A/B leads to a decrease in the secretion of exosomes from cancer	Li et al. (2014)
GTPase Rab 5	Melanoma	Elevated Rab5 levels can stimulate tumor cell migration	Silva et al. (2016)
GTPase Rab 7	Melanoma	Upregulated Rab7 is an early-acting driver of melanoma that is indicative of patient prognosis	Alonso-Curbelo et al. (2014)
Heat shock proteins 60	Colorectal cancer	Elevated expression of Heat Shock Proteins (HSPs) is indicative of an unfavorable prognosis in cancer cases	Campanella et al. (2015)
Heat shock proteins 70	Breast cancer	Elevated expression of Heat Shock Proteins (HSPs) is indicative of an unfavorable prognosis in cancer cases	Gobbo et al. (2016)
miR-105	Breast cancer	A candidate of regulator of cancer cell migration when upregulated expression	Zhou et al. (2014)
miR-10b	Breast cancer	Linked to the development of malignant traits when upregulated expression	Anfossi et al. (2014)
miR-1229, let-7a	Colon cancer	A candidate of up-regulator of primary colon cancer	Sugimachi et al. (2015)
miR-125b, 130b, 155	Prostatic carcinoma	Promotes the development of cancerous changes when upregulated expression	Abd Elmageed et al. (2014)
miR-135b	Myeloma	Markedly increased in exosomes derived from HR-MM and boosts the formation of endothelial tubes	Umezu et al. (2014)
miR-19a	Breast cancer	A potential biomarker when upregulated expression	Anfossi et al. (2014)
miR-210	Leukemia	Elevated in exosomes and amplifies endothelial migration and tube formation	Tadokoro et al. (2013)
miR-214	Ovarian cancer	A candidate of up regulator of ovarian cancer when upregulated expression	Taylor and Gercel-Taylor (2008)
miR-223	Breast cancer	Stimulates the invasion of breast cancer cells when upregulated expression	Yang et al. (2011)
miR-23b	Bladder cancer	A candidate of regulator of cancer cell migration when upregulated expression	Ostenfeld et al. (2014)
miR-29a	Lung cancer	Associate with the growth and metastasis of tumor when upregulated expression	Fabbri et al. (2012)
Programmed cell death 6-interacting protein	Colorectal cancer	It’s upregulated expression is a significant indicator of the metastatic process	Valcz et al. (2016)
Tumor susceptibility protein 101	Breast cancer	Help to assess the number of exosomes in tumors when upregulated expression	Yang et al. (2017)

A particularly noteworthy attribute of exosome-based diagnostics lies in their non-invasive nature. The isolation and analysis of exosomes from easily accessible bodily fluids, such as blood or urine, present a transformative approach for clinicians and researchers (Wang et al., 2014; Sohn et al., 2015). This minimally invasive method not only facilitates early cancer detection but also enables the continuous monitoring of cancer dynamics over time (Thind and Wilson, 2016). The escalating comprehension of exosomes and their cargo augurs well for revolutionizing cancer diagnostics and tailoring personalized treatment strategies. Researchers and clinicians are actively harnessing the potential of exosomes to usher in novel horizons in the detection and management of cancer (Patel et al., 2019).

The retrieval and scrutiny of exosomes from common bodily fluids represent a paradigm shift in cancer diagnostics, underscoring its pivotal role in advancing healthcare. This innovative methodology assumes particular significance due to its capacity for early cancer detection. The identification of cancers in their early stages holds immense clinical importance, as prompt recognition often correlates with higher treatment success rates and substantially improved patient outcomes (Hoshino et al., 2015; Hinestrosa et al., 2022). The accessibility of bodily fluids such as blood, urine, and saliva for exosome retrieval streamlines the diagnostic process, augmenting its potential impact on healthcare. The utilization of exosomes for early cancer detection heralds a promising era in medicine—one with the potential to transform the oncological landscape by saving lives through early intervention and enhancing treatment outcomes (Kalluri and LeBleu, 2020; Yu et al., 2021). As ongoing research continues to unravel the intricacies of exosomes, their role in advancing cancer diagnostics and personalized medicine is poised to play an increasingly pivotal role in shaping the future of oncology.

### 4.2 Monitoring disease progression for therapeutic targets

The isolation and analysis of exosomes offer a dynamic window into the complex landscape of cancer, as they can be collected at various stages of disease progression (Khan et al., 2012). This versatility provides invaluable insights into the entire continuum of cancer development, from its initial onset to metastasis and response to treatment. By examining the molecular cargo of exosomes over time, clinicians gain a nuanced understanding of how cancers evolve, adapt, and potentially resist therapy (Tang et al., 2020). This real-time monitoring becomes a critical tool in tailoring treatment strategies for individual patients. For example, in the early stages, exosomes may contain biomarkers indicative of tumor initiation, offering an opportunity for timely intervention (Jalalian et al., 2019). As cancer progresses, exosomes can carry information about metastatic potential, aiding in the prediction of disease spread (Dai et al., 2020). Moreover, tracking changes in exosome content during treatment can guide clinicians in optimizing therapeutic regimens, making them more effective and personalized (Corradetti et al., 2021). Ultimately, the ability to harness exosomes as dynamic indicators of cancer progression has the potential to revolutionize cancer care, leading to more precise and successful treatment approaches.

Beyond their diagnostic potential, exosomes emerge as direct therapeutic targets in the fight against cancer (Zhou et al., 2021). Exosomes offer a highly efficient method for delivering small molecules, proteins, and RNAs to target cancer cells. Additionally, manipulating exosomes derived from cancer cells provides a novel approach to disrupting key processes in tumorigenesis (Scavo et al., 2020). By inhibiting the release or interfering with the function of these exosomes, it becomes possible to impede critical facets of cancer progression (Moitra et al., 2011). For example, interfering with exosome release from cancer cells may curtail their ability to communicate with neighboring cells and create a conducive environment for tumor growth (Wu M et al., 2019). Additionally, targeting the specific functions of cancer-derived exosomes can hinder metastasis, potentially preventing the spread of cancer to distant organs (Tai et al., 2018). Furthermore, disrupting the exosome-mediated transfer of molecules that confer resistance to treatment can enhance the effectiveness of therapies (Steinbichler et al., 2019; Zeng and Fu, 2020). Generally, the therapeutic targeting of exosomes holds great promise for developing innovative strategies to combat cancer, offering hope for more effective treatments and improved patient outcomes.

In conclusion, exosomes represent a promising avenue for cancer research and diagnosis. Their unique ability to carry and transfer cancer-specific information opens new possibilities for early detection, monitoring, and personalized treatment strategies. As researchers continue to unravel the complexities of exosome biology and their role in cancer, we can anticipate groundbreaking advancements in cancer diagnostics and therapies in the years to come.

## 5 The limitations of exosome for drug application

While exosomes offer significant potential for drug application and delivery, they are not without their limitations, which need to be carefully considered in research and development. The key limitations of exosomes for drug application include:

### 5.1 Biological complexity and specific targeting

The intricate nature of exosomes, characterized by their diverse molecular cargo, poses significant challenges in harnessing them for precise drug delivery applications (McAndrews and Kalluri, 2019). Achieving consistent and effective drug loading into exosomes is a formidable task due to its inherent complexity (Mathieu et al., 2019; Kalluri and LeBleu, 2020). Ensuring that the therapeutic cargo is properly encapsulated within exosomes can be challenging, as it demands meticulous control over the encapsulation process to maintain therapeutic efficacy.

Furthermore, while exosomes possess a natural propensity to target specific cells or tissues, engineering them for precise targeting of disease sites adds a layer of complexity (He et al., 2022). The intricacies of achieving this level of precision involve modifying exosomes to recognize and bind exclusively to the intended target cells while minimizing off-target effects (Sun et al., 2020). Striking this delicate balance between specificity and avoiding unintended consequences remains a significant challenge in the development of exosome-based drug delivery systems (Chen H et al., 2021). Despite these challenges, ongoing research and innovation in exosome engineering hold great promise for advancing the field of targeted therapeutics.

### 5.2 Limited drug loading capacity and stability

Exosomes, while offering immense potential as drug delivery vehicles, do have limitations that must be considered in their application (Lee et al., 2023; Sadeghi et al., 2023). Their finite cargo capacity is one such constraint, which may pose challenges for drugs requiring high payloads (Jayaseelan and Arumugam, 2022; Malekian et al., 2023). Larger therapeutic molecules or those with low solubility might not be efficiently encapsulated within the limited confines of exosomes. This limitation necessitates careful selection of drugs to ensure compatibility with exosome-based delivery systems (Zhang L et al., 2021; Wu et al., 2022). Another concern is the fragility of exosomes. They can be susceptible to degradation, particularly when exposed to extreme temperatures or mechanical stress (Chen H et al., 2023; Zhang M et al., 2023). This fragility underscores the importance of maintaining proper storage and transportation conditions to preserve their integrity and functionality (Sanz-Ros et al., 2022). Ensuring stability throughout these processes can be a significant logistical challenge, but it is crucial for the successful deployment of exosome-based therapeutics.

In addressing these limitations, ongoing research focuses on optimizing exosome-based drug delivery by exploring innovative loading techniques, enhancing cargo capacity, and developing improved storage and transportation protocols. Despite these challenges, the versatility and potential of exosomes as drug carriers make them an exciting avenue for the future of targeted and personalized medicine.

### 5.3 Biological barriers and immunogenicity

When administered *in vivo*, exosomes encounter a series of biological barriers (e.g., including the blood-brain barrier, blood–cerebrospinal fluid barrier, blood–retinal barrier, and gastrointestinal tract) that must be overcome to effectively reach their intended target sites (Elliott and He, 2021). These barriers encompass the complex milieu of bodily fluids and tissues, which can hinder the distribution and efficacy of exosomes. Their journey through the circulatory system, extracellular matrix, and cellular barriers necessitates a thorough understanding of their interactions within these environments (Lakhal and Wood, 2011; Das et al., 2019).

Moreover, the immunogenicity of exosomes remains a topic of ongoing research and scrutiny. While they are generally considered to have low immunogenic potential, there are still gaps in our understanding (Zhao et al., 2022). Concerns about potential immune reactions to exosomes used for drug delivery persist. Undesirable immune responses could not only reduce the therapeutic efficacy of the delivered cargo but also trigger adverse effects in the recipient (Gyorgy et al., 2015). Therefore, meticulous investigation into the immune aspects of exosome-based therapies is essential for their safe and effective implementation in clinical settings.

### 5.4 Scale-up, production and cost

The large-scale production of exosomes with consistent quality presents significant challenges in terms of both technical feasibility and cost-effectiveness (Maumus et al., 2020). Establishing scalable manufacturing processes for exosome production continues to be a paramount concern within the field of exosome-based therapies. The complexity of isolating and purifying exosomes, coupled with the need for customization for specific therapeutic applications, can drive up production costs considerably (Sadeghi et al., 2023). The cost-intensive nature of exosome production has the potential to impact the affordability and accessibility of exosome-based treatments. As healthcare systems and patients alike seek cost-effective solutions, it becomes essential to develop more efficient and cost-efficient production methods (Ahn et al., 2022). This will not only make exosome therapies more financially accessible but also enable wider adoption within the medical community.

Recently, research endeavors have been dedicated to streamlining and optimizing exosome production processes, seeking ways to reduce costs without compromising quality, and ensuring that exosome-based treatments can reach a broader spectrum of patients in need. Achieving these goals will be pivotal in realizing the full therapeutic potential of exosomes.

## 6 The advantage of exosomes for cancer early detection

Studying exosomes can lead to the identification of novel biomarkers and therapeutic targets, advancing our understanding of cancer biology (Eassa, 2023). Importantly, Exosomes offer several advantages for cancer early detection, making them a promising avenue for improving the accuracy and effectiveness of diagnostic methods. For example, Exosomes can be collected at multiple time points, enabling dynamic monitoring of disease progression and treatment response (Chanteloup et al., 2020). Changes in the molecular cargo of exosomes over time can guide treatment adjustments, providing a real-time view of the disease (Sharma et al., 2017; Armstrong et al., 2018). The key advantages of using exosomes for cancer early detection include:

### 6.1 Specificity and high sensitivity

Exosome-based assays represent a significant advancement in cancer diagnostics due to their exceptional specificity and sensitivity (Salciccia et al., 2023). These assays are adept at detecting cancer-specific molecules, which greatly reduces the likelihood of false positives. This precision not only minimizes unnecessary diagnostic procedures but also alleviates the anxiety often associated with false alarms, ensuring a more patient-centric approach to the healthcare (Logozzi et al., 2023). Logzzi et al. reported that specific prostate-specific antigen (PSA) exosomes efficiently differentiated between prostate cancer (PC) and non-PC patients (benign prostatic hyperplasia (BPH) and healthy controls), outperforming the traditional serum PSA test. IC-ELISA achieved 98.57% sensitivity and 80.28% specificity in PC vs. BPH discrimination. Combining IC-ELISA with NFSC increased sensitivity to 96% and specificity to 100% (Logozzi et al., 2019).

What sets exosome-based assays apart is their capacity to detect even trace amounts of cancer biomarkers, even when they are present in low concentrations (Saad et al., 2021). This heightened sensitivity becomes a potent tool in the early detection of cancer, potentially identifying the disease before clinical symptoms manifest (Hu et al., 2022). This early detection, because of exosome-based assays, substantially enhances the chances of successful treatment outcomes, ultimately improving patient prognosis.

Moreover, exosomes offer a more comprehensive representation of the entire tumor’s molecular profile compared to traditional biopsies, where only a small portion of the tumor is sampled (Yu et al., 2021). This advantage significantly reduces the potential for sampling bias and ensures that clinicians have a more accurate and holistic understanding of the disease (Li L et al., 2023). In essence, exosome-based assays usher in a new era of cancer diagnostics, characterized by enhanced accuracy, sensitivity, and patient-centered care.

### 6.2 Non-invasive sampling and patient-friendly

Exosomes represent a groundbreaking avenue for non-invasive diagnostics as they can be conveniently isolated from readily accessible bodily fluids, including blood, urine, saliva, and cerebrospinal fluid (Lin et al., 2015; Yi et al., 2022). This non-invasive sampling method eliminates the need for painful and invasive procedures such as biopsies, which can be uncomfortable and entail inherent risks (Chen J et al., 2021). Consequently, exosome-based tests offer a patient-friendly approach, requiring nothing more than simple blood or urine collection (Liu et al., 2018). This simplicity in sampling not only enhances patient comfort but also encourages a broader spectrum of individuals to undergo regular screenings, contributing significantly to early cancer detection efforts.

Furthermore, the molecular cargo of exosomes can be meticulously analyzed to identify specific cancer types and discern their unique characteristics (Mitchell et al., 2022). This valuable information provides a foundation for the development of personalized treatment plans tailored to the patient’s specific cancer subtype (Srivastava et al., 2018). Recent studies demonstrated that exosomes from lung tumor cells reflected the genetic mutations of the parent cell lines, notably EGFR status (Thakur et al., 2014). In a separate *in vitro* study, exosomes from various cancer cell lines facilitated the transfer of activated EGFR to endothelial cells, triggering MAPK and AKT pathways (Al-Nedawi et al., 2009). Therefore, specific target methods can be developed in precise treatment strategies not only to improve therapeutic efficacy but also to minimize potential side effects, enhancing the overall quality of care and patient outcomes (Sancho-Albero et al., 2019; He et al., 2022). In summary, exosomes hold significant promise for cancer early detection due to their non-invasive nature, ability to detect cancer-specific biomarkers, high sensitivity, and potential for dynamic monitoring. Moreover, exosome-based diagnostics represent a transformative shift towards patient-friendly and personalized approaches to cancer detection and treatment.

## 7 Advanced strategies and technologies in exosome-based cancer detection

In the pursuit of harnessing the potential of exosomes to improve efficiency and precise cancer detection, several up-to-date innovative strategies and cutting-edge technologies have been developed (Chen J et al., 2021; Cheng et al., 2022). These approaches have the power to transform cancer diagnostics, providing more precise, non-invasive, and early detection methods. The key strategies and technologies that are paving the way for the utilization of exosomes in cancer detection.

### 7.1 Liquid biopsies for revolutionizing cancer diagnosis

Liquid biopsies are a groundbreaking strategy in cancer detection, utilizing the non-invasive collection and analysis of exosomes from body fluids like blood, urine, and saliva (Shegekar et al., 2023). This technique is a significant evolution from traditional tissue biopsies, which are often invasive and not always suitable for all patients. Exosomes, when released by cancer cells into the bloodstream, contain critical biomarkers (mutated DNA, proteins, microRNAs) that offer real-time insights into tumor status, are more convenient and authentic than the circulating tumor DNA (ctDNA) and circulating tumor cells (CTCs) (Heitzer et al., 2019; Yu et al., 2022). For example, the abundance of exosomes, with concentrations around 10^9 particles per milliliter in biological fluids, facilitates the collection of these vesicles. In contrast, only a few circulating tumor cells (CTCs) are present in 1 mL of blood samples (Cai et al., 2015). On the other hand, due to their lipid bilayer composition, exosomes inherently possess stability, enabling them to circulate consistently under physiological conditions, even within the rigorous conditions of the tumor microenvironment. This intrinsic biological stability facilitates the prolonged preservation of samples for the isolation and detection of exosomes (Yu et al., 2021). Therefore, liquid biopsies on exosomes may facilitate early cancer detection, potentially before symptoms appear, and allow continuous monitoring of disease progression and treatment response. This method enhances early detection capabilities and provides a less invasive alternative to conventional biopsies.

### 7.2 Nanotechnology for precision and sensitivity in detection

Nanotechnology has been instrumental in developing exosome-based biosensors and platforms for the sensitive detection of cancer-associated biomarkers (Mukhtar et al., 2021). Nanoparticles and nanomaterials are used to isolate exosomes from biological samples accurately, ensuring precise detection of cancer-related exosomal cargo. These nano-based methods increase the sensitivity of assays, enabling the detection of minimal biomarker levels, thus improving the accuracy of cancer diagnoses and aiding in identifying specific cancer types and molecular signatures. For example, Patolsky et al. developed a nanotechnology-based method for DNA detection, which has the potential for cancer diagnosis. They achieved this by integrating biotin labels into DNA replicas. These replicas were attached to magnetic particles, forming a nanodevice. This innovative approach enhances the accuracy of DNA-based detection in cancer diagnostics (Patolsky et al., 2003). Similarly, the research team led by Xu made significant advancements in the field of nanoparticle technology for cancer diagnosis. They created shell-engineered nanoparticles (NPs) that were coated with silver (Ag) and gold (Au). This coating substantially improved the sensitivity of PCR-based DNA detection methods which can be used in identifying cancer (Zhao et al., 2014).

### 7.3 Machine learning and bioinformatics for interpreting complex data

The vast and complex data from exosome profiling necessitate advanced analysis tools, where machine learning and bioinformatics play a crucial role (Chen J et al., 2023). Machine learning algorithms are adept at identifying patterns in large datasets, making them ideal for interpreting exosome profiles (Li J et al., 2023). Li et al. present a machine learning approach for non-invasive cancer diagnosis using exosome protein markers, achieving high accuracy in identifying cancer types with an advanced biomarker signature and sophisticated data models, marking a significant leap in early cancer detection methodologies (Li B et al., 2023). Wang et al. employed the Least Absolute Shrinkage and Selection Operator (LASSO) regression algorithm to construct a prognostic model based on differentially expressed genes (DEGs). The model’s predictive accuracy and sensitivity were validated through prognostic analysis and receiver operating characteristic curve analysis (Wang Z et al., 2023). These algorithms can differentiate between cancerous and non-cancerous samples, classify cancer subtypes, and predict treatment outcomes. Additionally, bioinformatics tools are essential for managing and interpreting the extensive data from exosome analysis, helping to derive meaningful insights from the complex molecular profiles of exosomes.

The integration of liquid biopsies, nanotechnology, machine learning, and bioinformatics represents a paradigm shift in cancer diagnostics. This convergence facilitates non-invasive, precise early cancer detection, and dynamic disease monitoring through exosome analysis. The precision of nanotechnology and the analytical power of machine learning and bioinformatics in interpreting complex data highlight the transformative potential of exosomes in cancer detection. As these technologies continue to evolve, exosome-based cancer detection is poised to become a fundamental aspect of early diagnosis and personalized cancer treatment, significantly impacting oncology by improving patient outcomes and expanding our understanding of cancer biology.

## 8 Prospective and conclusion

**FIGURE 3:**
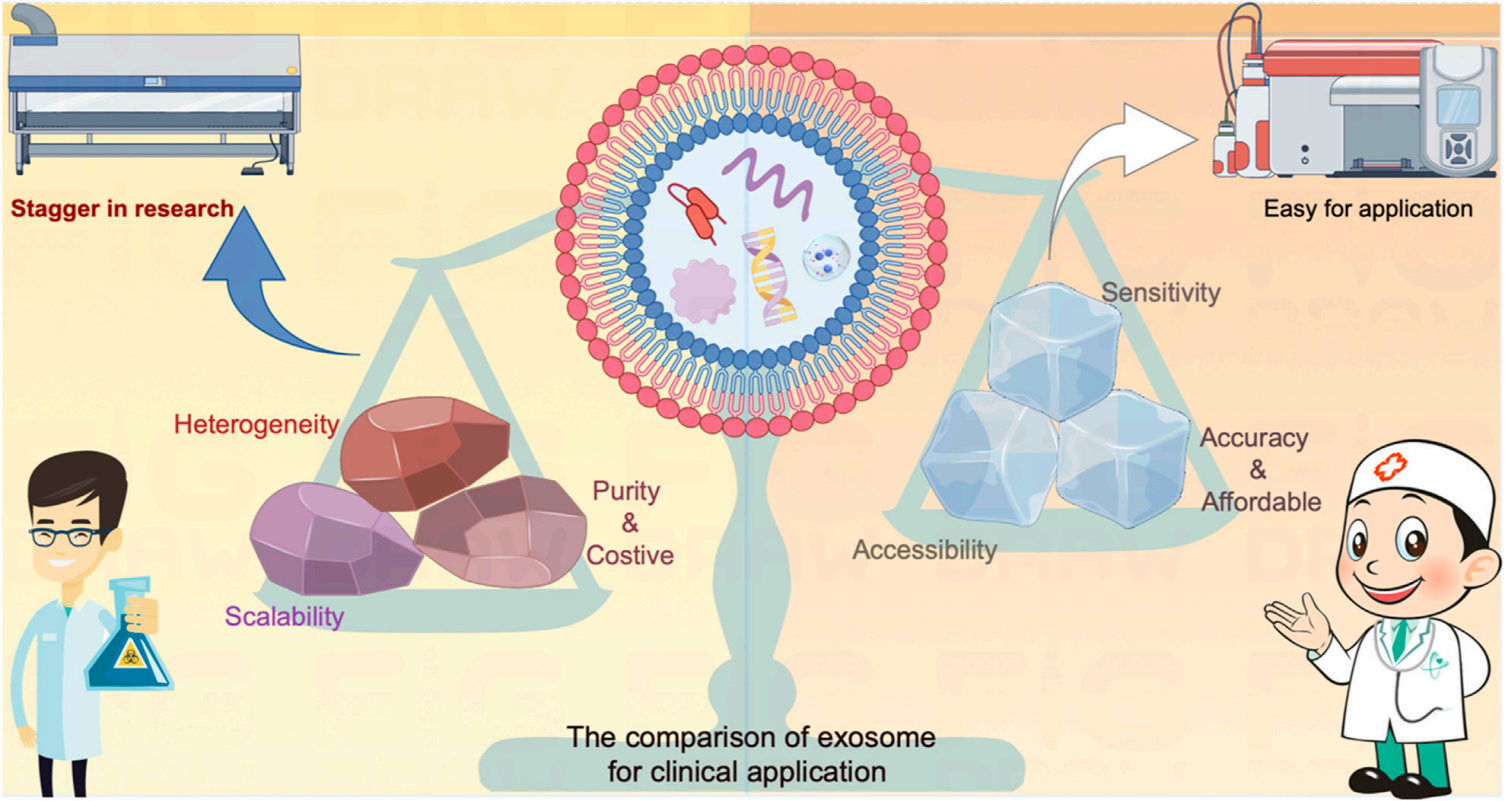
The comparison of exosome on diagnosis versus treatment for clinical application. The exosome serves as a carrier for various contents that actively influence cellular function or provide real-time insights into the developmental processes of target cells. These attributes have demonstrated significant value in the realm of cancer diagnosis and treatment. Nevertheless, the direct utilization of exosomes as mediators for cancer treatment faces inherent limitations due to contemporary technical challenges, encompassing issues related to heterogeneity, scalability, and purity. These constraints impede the seamless translation of exosome-based therapies from laboratory research to clinical applications. In contrast, leveraging exosomes as sensors offers a promising alternative. By repurposing them into highly sensitive, easily accessible, accurate, and cost-effective tools, these challenges can be transformed into opportunities for detecting cancers. The incorporation of state-of-the-art diagnostic equipment facilitates a more expeditious and practical application of exosomes in cancer diagnosis compared to treatment methodologies.

Exosomes hold immense potential for cancer detection and are more practicable for achieving compared to cancer treatment per the accessibility in the clinical applications (Figure 3). However, several critical challenges need to be confronted to fully harness their potential: A pressing concern is the absence of standardized protocols for exosome isolation, characterization, and analysis. The establishment of rigorous standards is imperative to ensure the reproducibility and reliability of exosome-based assays across various laboratories and therapeutic companies. Standardization efforts will bolster the credibility of exosome-based diagnostics. Moreover, the success of exosome-based assays hinges on achieving heightened specificity and sensitivity. The aim is to minimize false positives and false negatives, which can have significant clinical implications. This necessitates the development of more selective and reliable biomarkers, as well as advancements in detection technologies.

On the other hand, exosomes exhibit great promise in the research field which has a well-controlled environment, but their real-world utility as cancer biomarkers needs to be rigorously validated through large-scale clinical trials. These trials are essential to establish the relevance and efficacy of exosome-based diagnostics. Demonstrating their performance in diverse patient populations is a crucial step toward clinical application. In addition, the ethical dimensions of employing exosomes for cancer detection require rigid contemplation. Therefore, obtaining informed consent from patients, ensuring data privacy, and adhering to ethical guidelines are very important. Safeguarding patient rights and privacy is an ethical requirement in the development and application of exosome-based diagnostics for cancer.

In conclusion, exosomes represent a promising avenue for cancer detection because they are enriched with cancer-specific biomolecules, and offer non-invasive, sensitive, and specific avenues to identify cancer-related markers. As research continues to advance, exosome-based assays can hold the potential to revolutionize cancer diagnosis, facilitating earlier detection and improving patient survival and life quality. Nevertheless, addressing the challenges associated with standardization, specificity, sensitivity, clinical validation, and ethical considerations is significant to comprehensively realize the translational potential of exosomes for cancer detection in clinical practice.
